# Therapeutic developments for neurodegenerative GM1 gangliosidosis

**DOI:** 10.3389/fnins.2024.1392683

**Published:** 2024-04-26

**Authors:** Dorian Foster, Lucian Williams, Noah Arnold, Jessica Larsen

**Affiliations:** ^1^Department of Chemical and Biomolecular Engineering, Clemson University, Clemson, SC, United States; ^2^Department of Bioengineering, Clemson University, Clemson, SC, United States

**Keywords:** GM1 gangliosidosis, substrate reduction therapy, enzyme replacement therapy, gene therapy, clinical trials, neurodegeneration, lysosomal storage disease

## Abstract

GM1 gangliosidosis (GM1) is a rare but fatal neurodegenerative disease caused by dysfunction or lack of production of lysosomal enzyme, β-galactosidase, leading to accumulation of substrates. The most promising treatments for GM1, include enzyme replacement therapy (ERT), substrate reduction therapy (SRT), stem cell therapy and gene editing. However, effectiveness is limited for neuropathic GM1 due to the restrictive nature of the blood–brain barrier (BBB). ERT and SRT alleviate substrate accumulation through exogenous supplementation over the patient’s lifetime, while gene editing could be curative, fixing the causative gene, *GLB1*, to enable endogenous enzyme activity. Stem cell therapy can be a combination of both, with *ex vivo* gene editing of cells to cause the production of enzymes. These approaches require special considerations for brain delivery, which has led to novel formulations. A few therapeutic interventions have progressed to early-phase clinical trials, presenting a bright outlook for improved clinical management for GM1.

## 1 Introduction

Lysosomal storage disorders (LSDs) are genetic disorders caused by dysfunction or deficiency of a lysosomal enzyme (Platt, 2018). GM1 is a neurodegenerative LSD caused by the dysfunction of β-galactosidase (β-gal), responsible for the breakdown of GM1 gangliosides and other glycolipids (Brunetti-Pierri and Scaglia, 2008). GM1 gangliosides are abundant in the brain, as they are involved with neural development and neuroplasticity (Rha et al., 2021; Guo, 2023). The disease results from at least one of over 200 autosomal recessive mutations to the *GLB1* gene (Brunetti-Pierri and Scaglia, 2008; Rha et al., 2021), with the specific mutation related to the degree of β-gal dysfunction and, thus, severity of the disease (Morita et al., 2009; Rha et al., 2021).

GM1 has an incidence of 1 in 100,000–200,000 live births and is separated into subtypes based on patient’s age at onset (Brunetti-Pierri and Scaglia, 2008; Rha et al., 2021). Infantile GM1 (0–6 months) is the most common, severe, often diagnosed by a cherry red macular spot and fatal by 2–4 years of age (Brunetti-Pierri and Scaglia, 2008; Rha et al., 2021). Late infantile GM1 (7 months–5 years) is less severe, while adult-onset GM1 (6+ years) is the least severe. Along with the decreasing severity, phenotypic clinical presentation becomes less consistent with delayed symptom onset, although neurological symptoms are consistent. Despite longer life expectancies, late-stage disease forms are fatal.

Physiologically, the direct consequence of malformed or missing β-gal is the accumulation of undegradable GM1 ganglioside and substrates. Lysosomal accumulation increases throughout disease, with an increasing number of swollen lysosomes leading to neural cell death, although the mechanism of neurodegeneration is incompletely understood. Reviews looking at the pathophysiology of GM1 exist elsewhere (Brunetti-Pierri and Scaglia, 2008; Cho and Jin, 2021; Nicoli et al., 2021; Rha et al., 2021; Senarathne et al., 2023). Current treatment methods focus on restoring endogenous β-gal activity and/or clearing accumulated GM1 gangliosides to decrease the disease burden.

## 2 Treatment strategies

**Figure 1:**
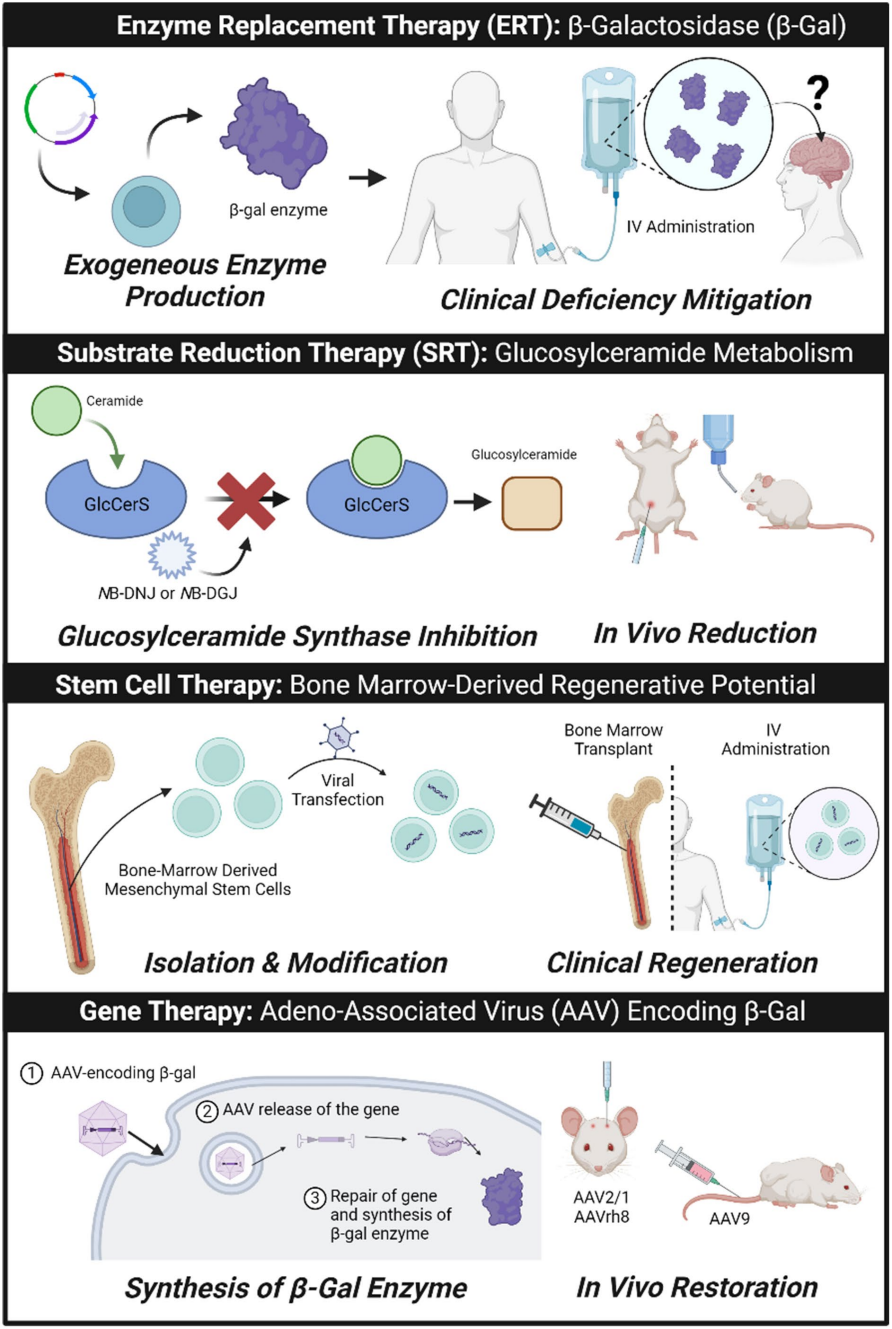
Treatment approaches currently being studied in pre-clinical and clinical settings for neuropathic lysosomal storage disorder GM1 gangliosidosis. Created with Biorender.

There is no treatment available for GM1, with current medications focused on symptom management. However, there are emerging therapeutic approaches that aim to replace, mimic, or assist primary β-gal functions (Figure 1); translation will require a major focus on bypassing the BBB or using more direct routes of administration to the central nervous system (CNS).

### 2.1 Enzyme replacement therapy

Perhaps the most translatable GM1 treatment approach is the exogenous replacement of the dysfunctional enzyme. ERT, wherein enzymes are intravenously (IV) administered weekly or biweekly indefinitely, has been clinically available for certain LSDs for over 15 years (Solomon and Muro, 2017; Concolino et al., 2018). However, in LSDs with clinically available ERT, symptomatic and prognostic improvements are observed only in somatic organs (Concolino et al., 2018). Free enzymes cannot cross the BBB; thus, the CNS is not helped by ERT, which stands as its primary limitation (Brunetti-Pierri and Scaglia, 2008; Concolino et al., 2018) for GM1. ERT does not rely on endogenous β-gal activity for therapeutic efficacy and therefore could be applied to all forms of GM1, if β-gal could be neurally delivered. For GM1, recombinant human β-gal has been produced by mammalian cells, plants, and yeast (Movahedpour et al., 2022), enabling its potential use in ERT. Pre-clinical models have demonstrated that even limited restoration (depending on subtype) of normal β-gal levels clears GM1 gangliosides and other stored substrates (Suzuki et al., 2009). Research to enable ERT as an option for GM1, therefore, centers on brain-targeted delivery approaches. One approach involves intracerebroventricular (ICV) administration, directly to the cerebrospinal fluid (CSF). ICV-administered recombinant human β-gal was able to successfully augment neurological β-gal and GM1 ganglioside levels to reverse brain pathologies in GM1-affected mice (Chen et al., 2020). However, ICV injections are invasive and high-risk. IV administration could significantly reduce the burden and risk on the patient, making clinical translation more likely, but will require transport through the BBB. Generally, these strategies are based on nanotechnology-mediated β-gal delivery, with targeting ligands enabling brain penetration (Kelly et al., 2017). Enzymes fused to antibodies for transferrin have recently demonstrated brain penetration in Mucopolysaccharidosis I mice (Kida et al., 2023), which could be translated for GM1.

Beginning in 1978, liposomes were used to deliver β-gal to GM1-affected fibroblasts, restoring enzyme activity up to ~70% normal (Reynolds et al., 1978). Kelly et al. used polyethylene glycol-b-poly (lactic acid) (PLA) polymersomes (PSs) tagged with apolipoprotein E (ApoE) to encapsulate 0.011 mg β-gal/mg PSs as GM1 ERT. ApoE enabled lysosomal trafficking and restoration of normal β-gal activity at a low dose of 0.175 mg/cm^2^ in GM1-affected fibroblasts (Kelly et al., 2017). Early pre-clinical trials demonstrate successful delivery of β-gal to the whole brain *in vivo* post IV injection in GM1-affected felines (Larsen, 2023), with a nine-fold increase in enzyme activity in the cerebellum. ApoE-modified hyaluronic acid-b-PLA PSs (Paruchuri et al., 2022; Foster et al., 2024), designed to release β-gal in response to upregulated hexosaminidase A (McCurdy et al., 2014), restored normal autophagic function in GM1-affected cells demonstrating promise toward ERT post BBB-delivery (Paruchuri et al., 2022). Arginase-responsive dextran sulfate/poly-L-arginine polymer capsules effectively delivered β-gal to affected mouse and human fibroblasts yielding a restoration to normal GM1 ganglioside levels in all cell lines at a dosage of 50 capsules/cell, but brain delivery was not assessed (Gupta et al., 2017). Beyond nanotechnology-mediated ERT, β-gal fusion proteins have demonstrated preclinical promise. Condori et al. fused β-gal with plant lectin ricin toxin B-subunit (RTB), which binds cell-surface glycolipids/proteins that contain galactose/galactosamine (Cho and Jin, 2021), leading to a reduction of GM1 ganglioside levels in GM1-patient fibroblasts (Condori et al., 2016). IV injections of β-gal: RTB in GM1-affected mice led to moderate increases in neural β-gal activity (<10% normal) compared to untreated controls (Weesner et al., 2022). Similarly, injecting β-gal fused with a mouse transferrin monoclonal antibody twice a week for 17 weeks in GM1-affected mice led to functional improvements, despite negligible neural β-gal activity or ganglioside clearance (Przybilla et al., 2021). ERT delivery methods that work for other neuropathic LSDs could be applied to GM1 and have been reviewed elsewhere (Augustine and Mink, 2013; Solomon and Muro, 2017; Del Grosso et al., 2022).

### 2.2 Substrate reduction therapy

SRT mitigates GM1 ganglioside levels to slow progression of GM1 by impeding ganglioside synthesis, slowing down the rate of catabolism. SRT for GM1 involves inhibition of ceramide glucosyltransferase (GlcCerS), the enzyme responsible for catalyzing the glycosphingolipid (GSL) biosynthesis pathway (Platt et al., 2003), converting ceramide to glucosylceramide. Iminosugars, including N-butyldeoxynojirimycin (*N*B-DNJ, commercially known as miglustat or Zavesca^™^) and N-butyldeoxygalactonojirimycin (*N*B-DGJ) (Platt et al., 1994a,b), have emerged as key candidates. These drugs can cross the BBB and have received clinical approval for other LSDs, increasing their promise for GM1 therapy (Andersson et al., 2004). However, SRT cannot degrade previously accumulated substrate and non-GSL substrates may continue to accumulate (Liu et al., 1999).

Early work used SRT to look at short-term treatment in β-gal^−/−^ neonatal mice using *N*B-DGJ. Mutant mice received intraperitoneal (IP) injections from day p-2 to p-5 with 600 or 1,200 mg/kg body weight. Treatment reduced ganglioside content by 19% and GM1 content by 36% with no significant side effects or apoptosis. Treatment did, however, induce body and organ weight loss (Kasperzyk et al., 2004). Next, they studied the effects from day p-9 to p-15 using IP injections of *N*B-DGJ in C57BL/6 J, healthy control, and β-gal^−/−^ mice, measuring total gangliosides and GM1 ganglioside content in the cerebrum-brainstem (C-BS) and the cerebellum. Ganglioside content in the C-BS and cerebellum was decreased by 16% in control and 19% in mutants and 22% in control and 21% in mutants, respectively. GM1 content decreased by 18 and 17% in the C-BS and cerebellum of control mice and 35 and 41% in mutant mice. No adverse effects were seen in the mice and sphingomyelin content was shown to be increased in both mice at both locations of the brain (Kasperzyk et al., 2005). More recently, Elliot-Smith et al. looked at the effects of both *N*B-DNJ and *N*B-DGJ using 3–4-week-old β-gal^−/−^ mice. Mice received treatment through diet at doses of 1,200 mg/kg body weight/day. Results showed modest lifespan increases (~3 months) with NB-DGJ therapy only, but NB-DNJ did lead to decreased neural GM1 ganglioside. Both treatments enabled functional improvements, with greater improvement with *N*B-DNJ (Elliot-Smith et al., 2008). These studies have demonstrated modest efficacy of SRT in reducing lysosomal storage in early-stage GM1-affected mice by inhibiting the GSL biosynthesis process using iminosugars.

### 2.3 Gene therapy

Gene therapy involves delivery of functional DNA to target cells. For GM1, the leading approach utilizes viral vectors, predominantly adeno-associated viruses (AAVs), to restore β-gal activity to its normal state with a single injection. Over the past two decades, preclinical studies have demonstrated notable advancements, extending lifespan, restoring β-gal activity, and reducing storage levels in both the CNS and peripheral organs in murine and feline models (Hosseini et al., 2023).

Broekman et al. used ICV injections of AAV2/1 encoding mouse β-gal into GM1-affected neonatal mice. Post-injection, histological analysis revealed high levels of β-gal throughout the brain. Biochemical quantifications revealed a 7–65-fold increase in GSL levels, varying for each region of the brain, compared to the wild-type control and a 1,000-fold increase compared to non-treated, GM1-affected mice (Broekman et al., 2007). Baek et al. used the same vector, bilaterally injected in the thalamus in 6–8-week-old GM1 mice. Histological assessment verified elevated β-gal concentration in key regions, including the retrosplenial, visual, somatosensory, and auditory cortices. Notably, a 50% reduction in GM1 gangliosides was observed in the spinal cord, corresponding to an increase in survival of up to 52 weeks (Baek et al., 2010). Weismann et al. delivered AAV9, a BBB-bypassing vector, via tail vein injections into 6-week-old GM1-affected mice. GM1 ganglioside content was reduced by 36–76% with significant clearance in the cerebrum, brainstem, and spinal cord. Analysis at 10 and 30 weeks revealed improved motor function and behavior and a significant increase in median survival (Weismann et al., 2015). Hinderer et al. explored ICV injections of AAVhu68 encoding *GLB1* in one-month-old mice. Two highest dose groups experienced consistent improvements in gait and survived to study endpoint (300 days). Post-injection β-gal serum levels were 10-fold higher in the highest dose and on par with the heterozygote control in the second-highest dose. β-gal activity increases were dose-dependent, but all groups saw significant improvements compared to untreated mutants (Hinderer et al., 2020).

While mice play a crucial role in unraveling pathogenesis and exploring therapies, they do not fully replicate human diseases (Hein and Griebel, 2003; Bradbury et al., 2013). Feline GM1 gangliosidosis serves as a noteworthy model recapitulating late-infantile or juvenile GM1 (Gray-Edwards et al., 2017). Gray-Edwards et al. treated GM1 felines through intracranial injections of AAVrh8 encoding feline β-gal. GM1 ganglioside levels in AAVrh8-treated felines were restored to normal, reaching ~3% of the levels observed in untreated counterparts. A longitudinal investigation extending to 5 years post-injection revealed significant alterations in the amounts of key sphingolipid metabolites, demonstrating the effectiveness of the AAVrh8 vector (Gray-Edwards et al., 2017). The same group injected the same vector via an alternative route, ICV to the CSF, at high and low doses into GM1-affected felines. Survival rates increased 7.5-fold, with higher dose felines still alive at publication. Four months post-injections, β-gal was detected in the brain and spinal cord with higher distribution around the injection site (Gray-Edwards et al., 2020). Most recently Gross et al. employed IV administration of AAV9-carrying feline β-gal in six 1-month-old felines, dividing them into long-term (humane endpoint) and short-term (16 weeks) cohorts. X-gal staining revealed widespread distribution of β-gal in the brain, corresponding to an average survival extension of 5.3 times in the long-term cohort. The short-term cohort exhibited near-normal GM1 ganglioside levels with no evidence of toxicity seen in the AAV-treated felines (Gross et al., 2022).

### 2.4 Stem cell therapy

Stem cell therapy, injecting genetically-modified or donor stem cells, has been therapeutic in nonneuropathic LSDs (Hoogerbrugge et al., 1995; Kelly et al., 2017), but has not gained much traction for GM1. In 2004, Alessandra d’Azzo’s group delivered bone marrow-transplanted hematopoietic stem cells from healthy mice (lentiviral-transfected to produce β-gal) to GM1-affected mice. Stem cells maintained a detectible presence over 6 months, with a second injection leading to some neural β-gal expression (Sano et al., 2004). Expansion of findings demonstrated that β-gal-expressing stem cells migrate to the brain using a chemokine gradient, slightly improving β-gal activity in the cerebellum and brainstem in GM1-affected mice compared to wild type. In targeted regions, GM1 ganglioside storage was decreased, which correlated with higher β-gal activity and motor function improvement in treated mice (Sano et al., 2005). Bone marrow transplantation was proven to be an ineffective route *in vivo* for GM1 gangliosidosis in 2005 when a 7-month-old with juvenile GM1 was treated pre-symptomatically with fully-matched familial donor cells via bone marrow transplant and no changes to disease progression occurred (Shield et al., 2005).

As an alternative, intracerebral transplantation of β-gal-expressing mesenchymal stem cells (MSCs) in GM1-affected mice did not graft and survive long-term (>8 weeks) with modest increases in neural β-gal activity compared to untreated animals (≤44%) at 4 weeks, and limited increases in activity (≤26%) after 8 weeks, corresponding to ~3% normal activity (Sawada et al., 2009). Recently, bone marrow-derived stem cells isolated from β-gal^−/−^ mice and transfected with a lentivirus encoding cGLB1 for transplantation led to significant increases in β-gal activity in the cerebrum, hippocampus, and cerebellum (up to 19.7% of normal mice) with corresponding decreases in storage products, including GM1 ganglioside. A secondary transplantation at 32 weeks post-transplantation may have assisted in maintenance of β-gal activity, although activity still declined. Findings were repeated in GM1-affected felines, where MSCs, transfected to produce human β-gal with a lentiviral vector, injected intracortically survived and spread throughout the brain, where they expressed β-gal for 6-weeks (study endpoint) (Martin et al., 2005).

### 2.5 Clinical trials for GM1

There are two clinical trials in progress centered around gene therapy (Table 1). Gene transfer vector AAV9/GLB1A is being assessed for safety and efficacy following single IV administration in patients with a biallelic mutation in the *GLB1* gene and a documented β-gal deficiency (NCT03952637). Patients must also present phenotypically consistent with respective GM1 subtypes, with exclusion for advanced disease progression, use of interfering therapies, or compromised immune system. Study participants (currently 17) are dosed with 1.5E13 or 4.5E13 viral genomes (vg)/kg and tracked over 3 years. Specific outcomes center on survival, neurological and motor function, and developmental changes. In the second study, an AAVhu68 serotype delivery mechanism for GLB1 gene, PBGM01, is being investigated for safety, tolerability, and efficacy in phase 1/2 clinical trials on Type I and IIa GM1-affected juveniles (NCT04713475) post cisterna magna injection. Optimal dosing per estimated brain weight is determined in Phase I, to be examined in an expanded cohort in Phase II. Patient inclusion criteria appear to be looser than NCF03952637, requiring a laboratory-confirmed diagnosis, with similar exclusion criteria. Outcomes include assessment of the number of adverse or serious events within the first 5 years after treatment as well as monitoring of the patient’s change in developmental milestones.

**Table 1:** A summary of human clinical trials (ongoing and historic) that have focused on therapy for GM1 gangliosidosis.

ID Number	Country	Therapy	Material	Administration route	Status	Findings	Sources
NCT03952637	United States	Gene therapy	AAV9-GLB1	Intravenous	Active	N/A	National Institutes of Health National Library of Medicine (2024)
NCT04713475	United States	Gene therapy	AAVhu68 viral vector	Intra-cisterna magna injection	Active	N/A	National Institutes of Health National Library of Medicine (2024)
NCT00176904	United States	Stem cell therapy	Hematopoietic stem cells	Stem cell transplantation	Completed (2010)	120/135 patients reached the 100 day mark 92/135 patients reached the 1 year mark 81/135 patients reached the 3 year mark	National Institutes of Health National Library of Medicine (2024)
NCT01626092	United States	Stem cell therapy	Hematopoietic stem cells	Stem cell transplantation	Completed (2013)	1/3 patients achieved engraftment at the 100 day mark	National Institutes of Health National Library of Medicine (2024)
N/A	Italy	Substrate reduction therapy	Miglustat	Oral	Completed (2017)	Patients 1 and 2—improved motor function, vocalizations, and attention Patient 3—autonomous ambulation and normal IQ	Deodato et al. (2017)
N/A	Italy	Substrate reduction therapy	Miglustat	Oral	Completed (2020)	Patient 1—improved kyphosis, decreased motor and language skills Patient 2—stable impairment Patients 3 and 4—severe decrease in cognitive, motor and attention abilities	Fischetto et al. (2020)
N/A	United Kingdom	Bone marrow transplantation	Healthy bone marrow transplantation (repopulation of parenchymal microglial cells)	Surgical bone marrow transplantation	Completed (2005)	Patient developed normally until 25 months of age where myelination issues became significant; at 5-year mark patient became ataxic; at 7-year mark patient became wheel-chair bound and seizure prone	Shield et al. (2005)

While not in clinical trials in the United States, SRT using daily oral Miglustat has progressed to human trials in Italy to assess patients via neurological examinations, motor function tests, as well as changes to signs and symptoms on the regimen (Deodato et al., 2017; Fischetto et al., 2020). The dosing regimens were either constant at 600 mg/day (Deodato et al., 2017) or relative to body surface area (Fischetto et al., 2020). Both studies reported gradual neurological improvements, although only evaluating seven patients. Miglustat was also tested in the United States (NCT02030015) for infantile gangliosidoses, including GM1, although ultimately unsuccessful, with death in 13 of 16 patients prior to termination in 2021.

GM1 has fallen within the inclusion criteria for a few wider-ranging trials designed to study hematopoietic cell transplantation as it applies to a collection of diseases, although it is unclear how many enrolled participants had GM1. One study (NCT00176904) included 135 participants who underwent chemotherapy and transfusion with stem cells possessing the dysfunctional enzyme limiting substrate degradation; grafting of stem cells was largely successful, but only 81 participants survived past 3 years. Another study (NCT01626092) administered the cell transplant to patients with lysosomal and peroxisomal disorders following pre-treatment with low-dose total body irradiation. The three study participants were assessed for engraftment success, mortality and neurological outcomes. Other clinical trials have been initiated in recent years but terminated before completion, including a gene therapy trial (NCT04273269), Miglustat as SRT (NCT02030015), and another testing umbilical cord-supported stem cell therapy (NCT00654433).

## 3 Future outlook

GM1 is currently a fatal neurodegenerative LSD, warranting increased resources and research to address stakeholder concerns. Patients never reach developmental milestones, even with extensive symptomatic care. Treatment development should involve a focus on (1) treatment of the brain for the entirety of the patient’s life (2), impact on patient lifestyle (3), maintenance of systemically high β-gal activity, and (4) safety profile of either single or repeated doses. With these targets in mind, many currently explored preclinical treatments are falling short. The only ongoing clinical trials in the United States involve adeno-associated viral vectors (AAVs). One trial involves invasive intra-CSF injections (cisterna magna) while one is non-invasive, using IV-injected AAV9 to bypass the BBB for neural gene editing. Although initially promising, AAV9 can be neurotoxic in animal models (Hinderer et al., 2018) and, therefore, could also be neurotoxic in patients. Simultaneously, due to the development of neutralizing antibodies against AAVs, patients can typically only tolerate a single injection (Gray et al., 2011). Therefore, therapeutic success is dependent on success of the primary injection. AAV treatment of GM1-affected felines, while treating neurologic manifestation, led to later-in-life systemic symptoms that were previously subclinical, as was seen in feline Sandhoff disease (Johnson et al., 2023). This may also be the case in patients treated in clinical trials with AAV9. Because of this, gene therapy approaches may be high risk-high reward options for GM1 patients, capable of correcting the disease if treatment is done early.

Stem cell therapy has been unsuccessful *in vivo* for GM1 and other neuropathic LSDs, and the ethical concerns make it challenging to continue. Independently, SRT or ERT are also complicated. SRT cannot degrade previously accumulated substrate, making early diagnosis and intervention significant. However, Italian clinical trials have demonstrated the potential of SRT in seven patients, and results should not be ignored. If β-gal can be delivered to the brain, ERT is widely considered to be the safest option among LSD treatments. However, anti-ERT antibodies will develop and infusion-adverse reactions are possible (Concolino et al., 2018). Additionally, there is a potential for regular β-gal delivery to disrupt innate protein/enzyme complexes necessary for cellular protection (Chen et al., 2020). Specifically, both ERT and SRT demonstrate major lifelong burdens to GM1-affected patients due to the need for repeated administration. An adjuvant approach is not often considered, with either ERT or SRT developed to support patients post-gene therapy. If ERT and/or SRT is successful, it could support an imperfect initial gene therapy injection or accommodate diminishing β-gal activity, leading to curative therapy for the life of GM1-affected patients.

## Author contributions

DF: Writing – original draft, Writing – review & editing. LW: Writing – original draft, Writing – review & editing. NA: Writing – original draft, Writing – review & editing. JL: Supervision, Writing – original draft, Writing – review & editing.
